# Traditional Latvian herbal medicinal plants used to treat parasite infections of small ruminants: A review

**DOI:** 10.14202/vetworld.2021.1548-1558

**Published:** 2021-06-17

**Authors:** Alīna Kļaviņa, Dace Keidāne, Renāte Šukele, Dace Bandere, Līga Kovaļčuka

**Affiliations:** 1 Institute of Food and Environmental Hygiene, Faculty of Veterinary Medicine, Latvia University of Life Sciences and Technologies, LV-3004, Jelgava, Latvia; 2 Department of Pharmaceutical Chemistry, Faculty of Pharmacy, Rīga Stradiņš University, LV-1007 Riga, Latvia; 3 Department of Pharmacy, Red Cross Medical College of Rīga Stradiņš University, LV-1007 Riga, Latvia; 4 Baltic Biomaterials Centre of Excellence, Headquarters at Riga Technical University, Dzirciema Street 16, Riga, LV1007, Latvia; 5 Clinical Institute, Faculty of Veterinary Medicine, Latvia University of Life Sciences and Technologies, LV-3004, Jelgava, Latvia

**Keywords:** antiparasitic, gastrointestinal nematodes, heather, mugwort, polyphenols, sheep, tansy, wormwood

## Abstract

Numerous treatment agents offering prophylaxis against livestock parasites are commercially available. However, because of increasing antiparasitic drug resistance, the increased popularity of environmentally friendly lifestyle choices, and organic farming, there is more demand for new alternatives to livestock anthelmintic control strategies and medications. It is important to develop antiparasitics that are safe, effective, inexpensive, and environmentally safe. Local, traditional herbal plants such as tansy, mugwort, wormwood, and heather may serve as treatments for intestinal parasites of sheep. This overview provides knowledge of traditional Latvian plants with antiparasitic activities to establish a database for further research to develop new herbal antiparasitic drugs.

## Introduction

Small ruminants, particularly sheep and goats, occupy an important ecological and economical niche in Latvian agriculture. Among 1.9 million inhabitants, there are 120,726 sheep and 3467 official sheep farms registered [1]. Sheep are kept in Latvia for numerous reasons. Most important, ruminants convert poor foodstuffs into important products such as milk, meat, and wool for human consumption [2]. Moreover, poor, overgrown, and wet fields serve as reservoirs for parasites of sheep such as *Fasciola hepatica*, *Paramphistomum* spp., *Heamonchus contortus*, *Trichostrongylus axei*, *Trichostrongylus colubriformis, Trichuris ovis*, and *Monezia expansa* [3] that cause widespread infections of sheep [4]. Diverse endo- or ectoparasites affect sheep; and their adverse effects on health, production, and welfare are robustly documented [5,6]. For example, *Trichostrongylidae* infection is among the major challenges to ovine health management because of deleterious disease-caused effects and emerging antiparasitic drug resistance to available manufactured veterinary anthelmintics worldwide [4,7].

Numerous prophylactic agents are commercially available. However, because of increasing resistance to anthelmintics and increased popularity of environmentally friendly lifestyle choices and organic farming, new alternatives to current parasite control and treatment strategies are urgently required. Therefore, medicinal products that are safe, effective, inexpensive, and safe for the environment must be identified. For example, newer treatment strategies employ locally harvested herbal plants. This modern branch of traditional ethnoveterinary pharmacology, similar to phytotherapy, is based on knowledge passed down through generations. This subdiscipline of pharmacology lost popularity during the intensive industrial pharmacological era; however, it is now regaining interest. Unfortunately, we lack compelling scientific evidence supporting the beneficial effects of herbal medications.

Here, we aimed to review the past and present status of traditional herbal medicinal products historically used in Latvian traditional (folk) medicine for their anticipated antiparasitic effects in comparison to the available scientific literature, particularly related to sheep. This overview attempts to consolidate scientific knowledge of traditional Latvian plants with antiparasitic activities to assist future research.

## Methods

We searched Scopus, Web of Science, EBSCO, and PubMed for relevant articles published between January 1980 and September 2020. Other publications were sourced from references in individual articles. Relevant articles were selected after reading their titles and abstracts, and full text was acquired if this information was insufficient to exclude the study. Five herbal plants were chosen for review.

## Parasitic Diseases of Sheep

Parasitic diseases of grazing production animals such as sheep and goats, commonly cause major loss of body weight and productivity leading to increased mortality, which represent important animal welfare concerns worldwide [8,9]. For example, mortalities of intensively stocked, untreated lambs during their 1^st^ year of life range between 10% and 45% [10]. Economic losses are caused by mortality, veterinary services, drug expenses, and production losses. Parasitic gastroenteritis is a prevalent parasitic disease of ruminants in all Baltic states and Europe [9,11-14].

Evidence indicates that *Trichostrongylidae* family nematodes are the most pathogenic main species of the genera *Haemonchus* and *Ostertagia*. *H. contortus* and *O. circumcincta*, which are the most frequently identified pathogens of the ovine gastrointestinal tract, cause numerous digestive disorders [15,16]. Both species employ a direct life cycle in which adults deposit eggs, which are excreted in the host’s feces into the environment. *Ostertagia* spp. and *Haemonchus* spp. females lay between 100-200 and 5000-15,000 eggs/day, respectively. Environmental temperature and humidity are important for the survival of eggs and the development of mature parasites. Further, *Trichostrongylidae* eggs survive colder climates [16,17]. After hatching, larvae undergo subsequent molts into infectious third-stage (L3) larvae that migrate horizontally (terrestrially) or vertically on grass stalks. Moreover, horizontal migration typically ranges from 5 m to 10 m [9,15,18].

Among larval stages (L1, L2, and L3), L3 larvae are the most resistant in the environment. For example, evidence indicates that L3 larvae survive for >6 weeks in soil, although gastrointestinal nematodes larvae possibly survive for 1 year under favorable environmental conditions [15,17,19]. Sheep are infected by ingestion of L3 larvae, which molt twice in the abomasum molt before maturation. Under favorable conditions, the lifecycle past 3 weeks. When animals ingest numerous L3 larvae at the end of summer, their further development may arrest for ≤6 months at L4 Adult nematodes are thin roundworms, ranging in length from 1.0 cm to 2.0 cm. *H. contortus* is usually red, while *Ostertagia circumcincta* is brown [15].

During the development of *O. circumcincta* larvae, the number of mast cells in the abomasum decreases, although the number of nonacid producing glands increases. Initially, changes occur only in glands where larvae develop. When the larvae reach 1.3-8 mm, the glands stretch, and the adjacent glands become affected, causing the mucous membrane of the abomasum to thicken and become hyperplastic. Sheep with a very high nematode load may develop gastric ulcers, which are accompanied by a predicted increase in the pH of the abomasum [15,20-23].

*H. contortus* feeds through sucking blood from the abomasum mucosa, which induces local inflammation of the mucosa at the parasite’s attachment site. An adult nematode can ingest 0.05 ml of blood per day, leading to anemia of the host. Further, a load of >10,000 adult nematodes in a lamb potentially causes fatal blood loss, and sheep may suffer from the toxic effects of metabolic end-products derived from larval and adult parasites [22,23].

In sheep, the main clinical signs of parasitic gastroenteritis are anemia, delayed growth, anorexia, and weight loss. Hypoalbuminemia frequently occurs in highly infested animals, contributing to edema of the lower jaw and diarrhea [9]. Abnormal gastric secretion occurs in highly infested sheep, which is characterized by increased levels of the secreted gastric hormone gastrin and the proenzyme pepsinogen (a proenzyme) in the systemic circulation [15]. Together, growth retardation, weight loss, clinical illness, veterinary services, treatment expenses, and lost production and income cause significant economic losses.

## Worm Control Practices in Ruminants

Numerous types of worm control practices are employed worldwide. For example, a diet supplemented with high amounts of protein, amino acids such as methionine and leucine, and rumen-protected protein boost immunity, inhibit parasite proliferation, maintain agricultural and commercial production, and reduce fecal egg counts during infection [24-26]. The commercial vaccine “*Barbervax*,” which was released in Australia, achieves efficacy in field trials; however, the production of its recombinant subunits was unsuccessful [27,28]. Copper oxide wire particles initially used as a mineral supplement are effective and safe for treating *H. contortus* infection of weaning lambs [29,30]. In Latvia and Baltic states, pharmaceutical deworming is the most frequently used management strategy.

Macrocyclic lactones, which are the main drugs currently used in veterinary practice to treat gastrointestinal helminths, include avermectins and milbemycins; benzimidazoles, including albendazole, febantel, and fenbendazole; tetrahydropyrimidines, including pyrantel and morantel; and cyclic depsipeptides, including emodepside, piperazines, and praziquantel [31]. The Latvian Food and Veterinary service drug register lists 658 prescription-only veterinary antiparasitic formulations, among which 21 are licensed as anthelmintic medications for treating sheep as follows: Ivermectin, levamisole, albendazole, closantel, and monepantel [32]. Ivermectin (a macrocyclic lactone), albendazole (a benzimidazole), or both are most frequently used by Latvian sheep farms.

Unfortunately, intensive use or overuse of anthelmintics, insufficient dosing, repeated treatment, and incorrect drug administration routes contribute to the development of selective pressure on the parasites. The increasing resistance of pathogenic gastrointestinal parasites to available anthelmintics is an important veterinary and economic concern of sheep and goat farms in numerous European countries [33-40], including Latvia’s neighbor Lithuania [11,35].

The rates of anthelmintic resistance in the lower temperature zones such as the Nordic countries, and possibly the Baltic states, are generally lower compared with those in warmer climates [6,41,42]. Unfortunately, anthelmintic resistance is directed against most classes of commonly used broad-spectrum anthelmintics. Resistance of gastrointestinal nematodes against benzimidazoles, imidazothiazoles, tetrahydropyridines, and macrocyclic lactones occurs in sheep [6,14,42-44]. A study using fecal egg-count reduction tests found that anthelmintic resistance in the Baltic state is 27.8% in Lithuanian sheep flocks [11,35]. Macrocyclic lactones, benzimidazoles, and levamisole were used to treat gastrointestinal nematodes in 68.6%, 27.5%, and 3.9% of cases, respectively. A pilot study conducted in Estonia detected resistance against benzimidazoles and macrocyclic lactones [45]. Unfortunately, data are not available regarding anthelmintic resistance in Latvian sheep farms, which inspired the present investigation.

## Plants Used in Traditional (Folk) Medicine in Latvia

Latvian culture retains a strong folk memory that frequently contributes to an understanding of veterinary pathogens. For example, knowledge of diverse home remedies and the medicinal properties of plants is historically orally communicated through families, communities, and between generations. Recent research shows the importance of such folk traditions. For example, a summary of local plants used in Latvia during the 19^th^ Century was obtained from the Archives of Latvian Folklore. The collection comprises songs, poems, and books with 1900 records on the use of medicinal plants. According to these records, 211 plant genera representing 71 families were used by indigenous Latvian people during the 19^th^ Century. Possible therapeutic uses of local plants and their components, including individual dosages and descriptions of routes of administration, are described in this research [46,47].

Although human medicine commonly employs plants, very little data are available regarding ethnoveterinary medicine in Latvia. For example, a study conducted in Europe did not include reports of herbal medicine applied to animals [48]. The geographical location of Latvia and its consequently temperate climate supports and promotes an abundance of diverse flora. An impressive variety of local plants is, therefore, available for use in herbal medicine, particularly in its veterinary subdiscipline.

Herbal drugs were extensively used worldwide as anthelmintics before the introduction of modern broad-spectrum pharmacological agents [49]. Further, certain modern anthelmintics are derived from naturally occurring plants or their synthetic analogs [50]. Research performed to develop effective drugs from herbal compounds [51-53] identified drugs subsequently shown to be economically efficient, with minimal adverse effects [52].

Here, we reviewed more than 128 scientific articles on ethnoveterinary medicinal plants used in countries that share Latvia’s representative flora. We selected and summarized plants with suspected antiparasitic/anthelmintic properties naturally occurring in Latvia. We identified 82 plant species and 32 plant families that met the inclusion criteria. The most widely represented plant family was *Asteraceae* – 18 species, followed by *Apiaceae* – 7, *Lamiaceae* – 6, *Fabaceae* – 4, *Ranunculaceae* – 4, and *Poaceae* and *Salicaceae* – 3 representatives each. Other families included one or two representatives. The *Asteraceae* family is the most common medicinal plant found outside of Latvia, with a similar distribution of families [48].

Despite a large number of plants with medicinal properties for human and veterinary medicine, these properties are greatly affected by the chemical composition of each plant, particularly by secondary plant metabolites. Such compositions may vary depending on climate, country, soil properties, season, and other environmental conditions. The fact that a plant has anthelmintic activity in one part of the word does not mean that it will be the same in another. The chemical composition and properties of a plant are affected by harvesting, storage, the components of a plant, and the type. Most frequently, the entire plant is used, or various combinations of plant extracts [54]. Further, it is important to choose the appropriate extraction solvent according to the solubility’s of secondary metabolites of a target plant. Water and methanol are most frequently used solvents. Some believe ethanolic extracts are better, because it is easier for them to enter the parasite’s body through transauricular absorption [3].

Two broad study types are used to determine a plant’s properties – *in vitro* and *in vivo*. Each study has advantages and disadvantages. *In vitro* studies are less expensive, simultaneously analyze multiple plants, and are reproducible [55]. Further, *in vitro* studies allow the investigation of specific parasites and their lifecycles. The egg hatch (eggs), larval development (larvae), and larval motility (adults) tests are typically performed. *In vivo* studies are lengthier, only investigate one plant at a time, and are difficult to replicate. An *in vitro* study should initially be performed, and the plants that exhibit the best effect should be selected for subsequent *in vivo* studies. Often, the *in vitro* and *in vivo* results for the same plant differ. Further, the outcomes can be influenced by an animal’s internal factors, and plant species exert clinical effects that vary depending on the digestive system of the host, for example, ruminant versus a monogastric animal [54-56].

Thirty plants (including trees and shrubs) with anthelmintic activity toward ruminant endoparasites are listed in Table-1 [57-71]. Four plants were studied and are discussed in detail. The tansy, wormwood, and mugwort are widely used in herbal medicine, and heather (*Calluna vulgaris*) is described less frequently. Research into newer natural substances for medicinal use is trending [72]. Secondary metabolites of plants such as alkaloids, steroids, phenolics, tannins, flavonoids, resins, and fatty acids, have important impacts on health and exert numerous medicinal effects. Thousands of extracted and separated natural compounds serve as pharmaceuticals. Many conventional drugs such as quinine, artemisinin, and morphine are derived from natural sources [73,74].

**Table-1:** Plants used for ruminants endoparasites.

Scientific name	English name	Botanic family	Parasites type	Used parts**	Animals*	References
*Achillea millefolium* L.	Yarrow	*Asteraceae*	Gastrointestinal nematodes	W, E	S	[58]
*Acorus calamus* L.	Sweet – flag	*Araceae*	Helminths	R	C	[58]
*Alnus glutinosa* L.	Alder	*Betulaceae*	Trematodes	Sh	S	[59]
*Artemisia absinthium* L.	Wormwood	*Asteraceae*	Nematodes (*Haemonchus contortus*, *Trichostrongylus colubriformis*, *Toxocara vitulorum*), Cestodes, Protozoa (*Eimeria* Spp.)	A, E, W, L	C, S, G	[57,59-62,64]
*Artemisia campestris* L.	Field wormwood	*Asteraceae*	Nematodes (*Haemonchus contortus*)	L, E	S, G	[69,62-64]
*Artemisia maritime* L	Sea wormwood	*Asteraceae*	Nematodes	-	S	[62]
*Artemisia vulgaris* L.	Mugwort	*Asteraceae*	Nematodes (*Trichostrongylus colubriformis*)	-	C, S	[62,64]
*Betula pubescens* Ehrh.	Downy birch	*Betulaceae*	Helminths, Cestodas	L, Ba, sap	S	[59]
*Calluna vulgaris* L.	Hill/heater	*Ericaceae*	Trematodes	-	S	[59]
*Cichorium intybus* L.	Chicory	*Asteraceae*	Gastrointestinal nematodes, Lungworm	W	C, D, G, S	[59,62,66,67]
*Consolida regalis* Gray	Forking larkspur	*Ranunculaceae*	Nematodes (*Trichostrongylus colubriformis*)	F	-	[64]
*Daucus carota* L.	Wild carrot	*Apiaceae*	Nematodes (*Trichostrongylus colubriformis*)	R	-	[63,64]
*Dryopteris filix-mas* L.	Male - fern	*Dryopteridaceae*	Nematodes (*Trichostrongylus colubriformis*), Trematodes (*Dicrocoelium* Spp., *Fasciola* Spp.), Cestodas (*Moniezia* spp.)	R	C, G, S	[59,62,64,68]
*Erigeron canadensis* L.	Canadian fleabane	*Asteraceae*	Nematodes (*Trichostrongylus colubriformis*)	A	-	[64]
*Humulus lupulus* L.	Hop	*Cannabaceae*	Helminths, Cestodes, Trematodes	W, R	S	[59]
*Iris pseudocorus* L.	Yellow iris	*Iridaceae*	Helminths	R	C	[59]
*Juniperus communis* L.	Juniper	*Cupressaceae*	Trematodes (liver flukes)	B, R	C, G, S C	[58,59,63]
*Lepidium sativum* L.	Garden cress	*Brassicaceae*	Helminths, Trematodes	W, S	S	[59]
*Lotus corniculatus* L.	Bird`s – foot – trefoil	*Fabaceae*	Nematodes (*Cooperia oncophora*, *Ostertagia ostertagi*) Lungworms (*Dictyocaulus eckerti*)	A, L, W	C, D	[62,67]
*Nigella sativa* L.	Garden fennel – flower	*Ranunculaceae*	Gastrointestinal nematodes, Tapeworms	Ex, S	S, G	[60,69]
*Pastinaca sativa* L.	Wild parsnip	*Apiaceae*	Endoparasites	A	C, S, G	[63]
*Pyrus communis* L.	Pear	*Rosaceae*	Nematodes	B	S	[59]
*Quercus robur* L.	Pedunculate oak	*Fagaceae*	Helminths	N	C	[70]
*Salix* Spp.	Willow	*Salicaceae*	Helminths, Cestodes, Trematodes	Ba, L	C, S	[59,71]
*Sambucus nigra* L.	Elder	*Caprifoliaceae*	Cestodes, Trematodes	B	S	[59]
*Senecio vulgaris* L.	Groundsel	*Asteraceae*	Cestodes	L	C	[59]
*Symphori-carpos albus* L.	Snowberry	*Caprifoliaceae*	Endoparasites	L	C, G, S	[63]
*Tanacetum vulgare* L.	Tansy	*Asteraceae*	Nematodes, (*Trichostrongylus colubriformis*) Cestodes, Trematodes	A, W, L, S	S	[59,64]
*Urtica dioica* L.	Common nettle	*Urticaceae*	Helminths, trematodes	W, S	S	[59]
*Valeriana officinalis* L.	Common valerian	*Valerianaceae*	Nematodes (*Trichostrongylus colubriformis*)	R	-	[64]

* Animals: C=Cattle, D=Deer, G=Goats, S=Sheep.

** Used parts: A=Aerial parts, B=Berries, Ba=Bark, E=Extract, F=Flowers, L=Leaves, N=Nuts, S=Seeds, Sh=Shoots, W=Whole

Polyphenolic compounds, particularly flavonoids (>4000 structurally distinct molecules), are intensely studied secondary metabolites. The basic aglycone moiety comprises two benzene rings linked through a heterocyclic pyran ring. Flavonoids, which are often variably hydroxylated at various positions, are classified as flavones (luteolin, apigenin), flavonols (quercetin and kaempferol), flavanones (hesperetin), isoflavones (genistein), flavan-3-ols (catechin and epicatechin), and others [75]. The benefits of quercetin, catechin, kaempferol, resveratrol, apigenin, and luteolin are robustly documented. Flavonoids possess antioxidant, anti-inflammatory, antiseptic, antibacterial, and antiparasitic activities that protect against numerous chronic diseases and delay aging [74,76,77].

Polyphenolic tannins, which are of considerable medical interest as well, possess an astringent taste and are used in the food and pharmaceutical industries. Tannins are classified as hydrolyzable (gallic acid and ellagic acid) or condensed (proanthocyanidins). The medicinal properties of tannins are as follows: Anthelmintic, antibacterial, antidiarrheic, antiviral, antihepatotoxic, antihypertensive, anti-ulcer, anticancer, and antioxidant (condensed tannins only) [43,78,79].

Alkaloids are heterocyclic nitrogen-containing compounds, representing an undoubtedly important group of secondary metabolites. Approximately 20,000 alkaloids have been isolated, mainly from plants, but occur as well in microorganisms, marine organisms, and terrestrial animals. Alkaloids are classified according to their molecular backbones or according to their botanical origins. The most important functional groups include indole alkaloids, isoquinoline alkaloids, tropane alkaloids, steroidal alkaloids, pyridine, and pyrrolizidine alkaloids [80]. Pharmaceuticals derived from natural alkaloids include commonly used analgesic and cardiovascular agents, chemotherapeutic and antimalarial agents, centrally acting sedatives and stimulants, smooth muscle relaxants, and others. Important examples include atropine, cysteine, scopolamine, cocaine, quinine, morphine, codeine, ephedrine, reserpine, ergotamine, vinblastine, vincristine, and caffeine [76,79,81-83].

Several clinical trials of extensively studied essential oils are in progress. These compounds comprise mixtures of volatile, complex hydroalcoholic oil-soluble compounds, which are used as cosmetics and pharmaceuticals. Their structures are as follows: Acyclic and monocyclic monoterpenes (1,8-cineole, alfa pinene, camphor, linalool, nerol, and geraniol), bicyclic and tricyclic monoterpenes (sabinene, thujone, and tricyclene), sesquiterpenes (e-nerolidol, carvacrol methyl ether, germacrene B, and cadalene), isoprenoides, and terpenoids that include aldehydes, ethers, alcohols (carvacrol, thymol, eugenol, and borneol), and sulfur- or nitrogen-containing compounds (diallyl disulfide and indole) [84,85]. Mentions of essential oil plants, essential oils, and their derived single constituents appear in pharmacopoeias and traditional medicine paradigms of different cultures. These substances have been used in medicine because of their potential antimicrobial, antiparasitic, antioxidant, anti-inflammatory, and anticancer effects, as well as to treat stress, anxiety, and depression [86-88].

*Tanacetum vulgare* L., or tansy, is a perennial herb produced by genera of the *Asteraceae* family. The plant has a specific smell; and wild tansy, which is widely distributed across Latvia, is commonly mentioned in Latvian folk medicine. Tansy exerts healing effects, including anti-inflammatory, antioxidant, antibiotic, and cytotoxic effects [88,89]. *Tanacetum balsamita* L. (customary), *Tanacetum parthenium* (L.) Sch. Bip. (syn. *Pyrethrum parthenium* (L.) Sm., or feverfew are cultivated in Latvia. Despite its use as a typical folk medicine plant, there are few scientific articles or studies that focus on tansy’s antiparasitic effects. For example, *T. vulgare* is ineffective on the eggs and larvae of *H. contortus* [90].

Tansy is rich in essential oils as revealed by gas chromatography-mass spectrometry analysis (49-55 volatile compounds). Combinations of compounds vary between samples and are affected by different growing locations and conditions. The yields of essential oils from tansy range between 0.35 and 1.90% (v/w). Commonly studied essential oil components are as follows: 1,8 cineol, alpha-pinene, beta thujone, borneol, Artemisia alcohol, camphor, caryophyllene, acetals, tanacetin, and tanacetol A, B. *T. vulgare* essential oil contains a high concentration of thujone (a and b) (>50%) [91-94].

Thujone belongs to the essential oil group of isoprenoids (monoterpene ketone). High doses of thujone cause neurotoxicity in animals, manifested as hyperactivity, tremors, and tonic seizures. Therefore, it is best to use aqueous extracts of tansy, which contain lower doses of thujone [57]. Tansy flowers are used, for example, as antioxidants, antibacterial, anthelmintics, cytotoxic drugs, and repellents [93,95-98].

Recent studies show that tansy comprises numerous polyphenols. Spectrophotometric analysis of tansy flowers reveals gentisic, caffeic, chlorogenic, p-coumaric, and ferulic acids; hyperoside, isoquercitrin, rutin and quercitrin, patuletin, luteolin, kaempferol, and apigenin [89]. The total concentrations of phenol range from 46 mg to 50 mg gallic acid equivalents (GAE)/g. Polyphenolic compounds (phenolic acids) extracted from tansy possess antioxidant activities, although the antibacterial activities of its essential oil are low [99,100]. Further, methanol is the best extractant for polyphenols, compared, in descending order, with ethanol, water, isopropanol, and acetone. The yields of phenolics range from 12 mg to 63 mg GAE/g of the dried herb. Water serves as the most efficient extractant for preparing flavonoids [101].

*Artemisia vulgaris* L., or mugwort, is a very large (50-170 cm tall) herbaceous plant. Another widely distributed mugwort of the *Asteraceae* family grows in diverse habitats in Latvia. The plant possesses antileishmanial, antimalarial, antibacterial, antifungal, anthelmintic, and anti-inflammatory properties [102-104]. Mugwort comprises compounds with significant chemical polymorphisms and contains terpenes, a monoterpenoid, which contributes to the plant’s odor, which is the basis of its insecticidal activity, therefore, employed as an insecticide [105,106].

*Artemisia* is extensively studied, and numerous medicinal plants are derived from species of this genus. A review of different plants from this genus confirms their high concentrations of tannins and condensed tannins, which contribute to their antioxidant effects [107]. Artemisinin is a sesquiterpene lactone, which is produced by the *Artemisia* genus, is important for treating malaria [108]. Studies of the antibacterial effects of essential oils of *Artemisia* show that 1,8-cineole and alfa-thujone in the oil isolated from the aerial components of mugwort achieve good and nonselective activity against *Escherichia coli, Salmonella* Enteritidis*, Pseudomonas aeruginosa, Klebsiella pneumonia, Staphylococcus aureus, Candida albicans*, and *Aspergillus niger*. The diminished activity of essential oil from *A. vulgaris* root is likely explained by its reduced content of 1,8-cineole and thujone. *A. absinthi* essential oil contains a higher content of beta thujone and achieves higher antimicrobial effects, except when used to treat infections caused by *S*. *aureus* or *E. coli*. [109].

*A. vulgaris* is used in traditional medicine, and its herbs, leaves, shoots, roots, and essential oils are the subjects of numerous studies. GC/MS analysis revealed 24 different essential oil components [109] such as 1,8-cineole, alfa, beta thujone, camphor, borneol, beta-pinene, myrcene, and nerol [99,109]. The aerial components of *A. vulgaris* contain polyphenolics (flavonoids and tannins) and saponins [110]. High-performance liquid chromatography (HPLC) analysis shows that luteolin and morin are the main polyphenolics of methanol extracts of *A. vulgaris* [111], although hydroalcohol extracts, analyzed using HPLC/diode array detection, contain rutin, hydroxybenzoic acid, and caffeic acid [112].

*A. absinthium* L., or wormwood, which belongs to the *Asteraceae* family, similarly emits a specific wormwood odor, is widespread throughout Latvia; and folk medicine considers wormwood to have medicinal effects. Leaves and the aerial components of the plant are most commonly used in Europe [47,49]. *Artemisia* spp. found in Latvia are as follows: *A. campestris* L. (field wormwood), *A. annua* L. (annual mugwort), *A. austriaca* Jacq., *A. maritima* L. (sea wormwood), *A. sieversiana* Ehrh. ex. Willd., and *A. verlotiorum* Lamotte (Chinese mugwort). These rarely occur Latvia. In contrast, *A. dracunculus* L. (estragon) is the only one among these species that is cultivated as a herb and does not grow in the wild. *A. abrotanum* L. possesses medicinal properties.

*A. absinthium* serves as a source of numerous conventional and traditional medicines [113]. The most important chemical constituents in wormwood include flavonoids (santonin) and sesquiterpenes (artemisinin), which exert antiparasitic activities [105,114]. Artemisinin is a “parent drug” of antimalarial agents, and its derivatives have been used since 1980 [104]. These products are toxic to animals and induce neurotoxicity at high doses. *A. absinthium* essential oil contains thujone (a and b) and exerts antimicrobial and antifungal effects [3]. *A. absinthium* oil possesses antispasmodic, antiseptic, antiparasitic, and insecticidal properties [114,115]. Aqueous and ethanolic extracts of *A. absinthium* are effective against gastrointestinal nematodes in sheep, particularly against *H. contortus* [57]. However, a recent study of wormwood as a dietary supplement did not detect a significant effect on lambs infected with *H. contortus* [115]. A partial antiparasitic effect was detected in ruminants infected with *Eimeria* spp. [57].

Studies of the chemical composition of *Artemisia absinthium* identified and characterized different groups and individual active components of plants and essential oils. The essential oil, in minimal amounts, is detected using macro-and microscopic tests, assessed as absinthin and resorcinol [116]. The essential oil of *A. absinthium* contains 1, 8 cineol, camphor, linalool, alpha- and beta thujone, 4-terpineol, borneol, a-cardinol, absinthin, artemetin, myrcene, and other mono- and sesquiterpenes. Its essential oil is used as an anti-ulcer drug, an anticarcinogenic agent, an analgesic, an immunomodulator, an antimalaria, an antiseptic, an antibacterial, an anthelmintics, a larvicide, and a pesticide, although it may be toxic under certain circumstances [99,113,117].

Diverse polyphenolics are found in *A. absinthium*. For example, wormwood possesses a total phenolic content (TPCs) of 19±0.16 mg GAE/g [118]. Phenolic acids (tannic, gallic, salicylic, hemihydrate chlorogenic, caffeic, vanillic, syringic, ferulic, p-coumaric, rosmarinic, and trans-cinnamic acid) are detected in *A. absinthium*. The wormwood herb contains high levels of condensed tannins (proanthocyanidins), which contribute to its antioxidant activities. Flavonoid compounds include eupafolin, diosmetin, rhamnetin, apigenin as well as their glucosides luteolin, quercetin, rutin, and vitexin [99,119]. The concentrations (mg/g routine equivalents) of flavonoids and flavanols in wormwood extracts are 7.96±0.76 and 3.4±0.0, respectively [120]. The type of extractant affects the yields of flavonoids extracted from the wormwood herb. Ethanolic extracts contain higher amounts of phenolics than aqueous or chloroform extracts [121], which should be considered when preparing wormwood extracts for medicinal use.

*C. vulgaris* (L.), hill or heather, is a perennial plant of the *Ericaceae* family that grows in nutrient-poor soils and is often found in pine forests. In Latvia, heather, which is a valuable nectar-producing plant with medicinal properties, is the only species of the genus that grows in Latvia. The main chemical components of the plant that potentially engage parasites are tannins. *C*. *vulgaris* (L.) has low nutritional value, and only goats use it as forage. When goats were fed heather, only a mild anthelmintic effect is observed. Reduced numbers of released *T. colubriformis* egg are found, although heather increases immunity to parasites [119]. When heather is fed to experimentally infected goats, it is associated with a reduction in the establishment of *T. colubriformis* larvae and a significant decrease in nematode egg excretion by the host [122]. The aerial components of heather have been studied during different growth stages; however, heather’s medicinal properties are mainly described in traditional medicine, the plant’s pharmacologic potential is a focus of current investigations.

Phenolic compounds are found in varying amounts throughout *C. vulgaris*. The highest total concentrations of phenolic compounds, tannins, flavonoids, hydroxycinnamic acids, and proanthocyanidins are detected in leaves and roots during all growth stages, except during flowering. During flowering, the highest concentrations of flavonoids, proanthocyanidins, and anthocyanins are detected in flowers. Heather herb possesses antioxidant activity, and its antibacterial effects are mediated by the leaf, stem, rhizome, and root extracts. Leaf and stem extracts inhibit the infectivities of Gram-positive and Gram-negative bacteria, and roots are active against *Bacillus subtilis*, and extracts of the rhizome are active against *E. coli* [122]. Further, combinations of the active substances and amounts differ according to the season and are higher during growth. The total tannin concentrations in heather range from 22 mg to 36 mg TAE/g dw, with a higher proportion of the condensed form versus the hydrolysable form [123].

Aqueous extracts of inflorescences of heather contain carbohydrates, protein, lipids, fructose, glucose, organic acids, and fatty acids. Phenolic compounds identified in *C. vulgaris* extract are myricetin-3-O-glucoside and myricetin-O-rhamnoside. More polar extracts with more phenolics exhibit stronger antioxidant and antibacterial effects, whereas n-hexane and ethyl-acetate extracts achieve better anti-inflammatory and cytotoxic effects [124]. Liquid chromatography-mass spectrometry and ^1^H-nuclear magnetic resonance analyses of heather identified the fatty acids as follows: Linolenic (35%), linoleic (27%), and palmitic (21%); a high content of fiber and carbohydrates (75%); α-tocopherol quercetin, kaempferol, and myricetin derivatives as well as procyanidins. The total amounts of phenolic compounds (TPCs) are higher in hydroalcoholic extracts. These extracts exert good antibacterial effects without causing adverse effects [125]. Photospectrometric analysis (Folic–Ciocalteu) revealed the presence of TPCs at concentrations ranging from 67.55 mg GAE/g to 142.46 mg GAE/g and flavonoids at concentrations ranging from 42.11 RUE/g to 63.68 mg RUE(Rutin equivalent)/g (using aluminum chloride) [125]. Another study demonstrates antioxidant properties [126]. The phenolic compound quercetin likely accounts for heather’s calming effects [127,128].

## Conclusion

The emergence of helminths resistant to the most frequently used antihelmintic drugs requires urgent action to identify alternative methods. Herbal plants have huge potential for this purpose, as they are relatively inexpensive, safe, and effective. We, therefore, focused here on the typical Latvian herbal plants tansy, wormwood, mugwort, and heather as the focus of further *in vitro* studies to determine the effects of these plant extracts on strongyloid parasites.

## Authors’ Contributions

LK: Conceptualized the aim of the study, designed, supervised, and corrected the manuscript. AK, DK, RS, DB, and LK: Equally conceived the work with literature and drafted and reviewed the manuscript. All authors have read and approved the final manuscript.
